# Oxidative Stress, Neuroinflammation and Mitochondria in the Pathophysiology of Amyotrophic Lateral Sclerosis

**DOI:** 10.3390/antiox9090901

**Published:** 2020-09-22

**Authors:** Elena Obrador, Rosario Salvador, Rafael López-Blanch, Ali Jihad-Jebbar, Soraya L. Vallés, José M. Estrela

**Affiliations:** Department of Physiology, Faculty of Medicine and Odontology, University of Valencia, 15 Av. Blasco Ibañez, 4601016 Valencia, Spain; elena.obrador@uv.es (E.O.); rosario.salvador@uv.es (R.S.); loblanch@alumni.uv.es (R.L.-B.); aji.jebbar@gmail.com (A.J.-J.); lilian.valles@uv.es (S.L.V.)

**Keywords:** amyotrophic lateral sclerosis, motor neuron disease, neuroinflammation, oxidative stress, mitochondria

## Abstract

Amyotrophic lateral sclerosis (ALS) is a progressive motor neuron (MN) disease. Its primary cause remains elusive, although a combination of different causal factors cannot be ruled out. There is no cure, and prognosis is poor. Most patients with ALS die due to disease-related complications, such as respiratory failure, within three years of diagnosis. While the underlying mechanisms are unclear, different cell types (microglia, astrocytes, macrophages and T cell subsets) appear to play key roles in the pathophysiology of the disease. Neuroinflammation and oxidative stress pave the way leading to neurodegeneration and MN death. ALS-associated mitochondrial dysfunction occurs at different levels, and these organelles are involved in the mechanism of MN death. Molecular and cellular interactions are presented here as a sequential cascade of events. Based on our present knowledge, the discussion leads to the idea that feasible therapeutic strategies should focus in interfering with the pathophysiology of the disease at different steps.

## 1. Introduction

Amyotrophic lateral sclerosis (ALS), the most common motor neuron (MN) disease, is considered a rare disease based on its prevalence (2–3 cases per 100,000 people with European ancestry). The mean age of onset is 50–65 years, with only 5% of cases manifesting at 30 years or younger. In the younger patients, male predominate [1]. Mean survival from the first symptom is 3–5 years, although about 10% of patients live longer than 10 years [2].

The cause is unknown in 90–95% of all cases (sporadic ALS (SALS)), and just 5–10% of cases are associated with known genetic mutations (familial-type ALS (FALS)) [3]. More than 30 different genes have been linked to FALS [4]. However, the most common mutations are found in *C9orf72, SOD1, FUS,* or *TARDBP.* These represent approximately 40%, 20%, 1–5%, and 2–5% of FALS cases, respectively [5].

In the absence of a family history of the disease, there is no known cause of ALS. Nevertheless, the involvement of many possible causal/risk factors has been suggested. This includes (but not limited to) chemicals, metals, radiation/electromagnetic fields, physical activity, dietary habits, viruses, bacteria, prions, fungi, protein-related abnormalities, mitochondria-related pathology, glutamate excitotoxicity, higher energy expenditure than intake and progressive impairment of glucose metabolism, microtubule mediated deficits in axonal transport, reactive phenotypes in astrocytes and microglia, autoimmunity, and moderate to severe traumatic brain injury [3,6,7,8,9,10,11,12,13,14]

ALS is characterized by the progressive degeneration of cortical, brainstem, and spinal MNs. The region initially affected is typically within the upper limb, lower limb or bulbar musculature, however the subsequent progression is highly variable. Limb-onset is predominant (~70% of all cases) and is associated with the death of upper MNs (UMNs) in the brain, and lower MNs (LMNs) in the brain stem and spinal cord. Bulbar-onset (~25% of all cases) first affects the muscles in the face, mouth, and throat since MNs in the brain stem start to die first along with other LMNs. In the remaining 5% of all cases muscles in the trunk of the body are affected first. Yet many patients, even in late stages, can still move their eyes and sometimes control the sphincter and a few other muscles. Thus suggesting that some MNs are more vulnerable than others [15].

Common symptoms include muscle weakness, twitching, and cramping, leading eventually to muscle impairment. In the most advanced stages, patients will develop symptoms of dyspnea and dysphagia. Most patients die from respiratory failure [16].

The underlying mechanisms, as well as the cause or causes, of this devastating disease remain largely unknown. Consequently, ALS is designated by the FDA as an orphan disease.

## 2. Neuroinflammation and Oxidative Stress

Neuroinflammation, characterized by the presence of reactive astrocytes and microglia, moderate infiltration of peripheral immune cells, as well as elevated levels of inflammatory mediators, affects motor regions of the central nervous system (CNS) in SALS and FALS [17]. Microglia represents the primary form of active immune defense in the CNS [18]. If these cells cannot eliminate a toxic insult, they remain reactive and continue to recruit astrocytes and oligodendrocytes causing an ongoing inflammatory process [19]. In ALS patients and animal models, the abnormal proliferation of astrocytes (astrogliosis) that surrounds the degenerating MNs has been observed [20]. Reactive astrocytes in ALS express inflammatory markers, including cyclo-oxygenase-2, inducible NOS, and neuronal NOS [20], and inhibitory molecules that block regrowth of a damaged axon [21]. Moreover, astrocytes derived from the spinal cord of SALS or FALS patients have been shown cytotoxic to MNs in culture [22]. Furthermore, in mouse models of ALS, mast cells and neutrophils have been shown to accumulate around motor axons in the extensor digitorum longus muscle, sciatic nerve, and ventral roots, indicating that immune cell infiltration has far-reaching consequences along the entire peripheral motor pathway [23].

Remarkably, aberrant glial cells exhibit exacerbated endoplasmic reticulum stress together with a significant abundance of autophagic and secretory vesicles, all characteristic features of cellular stress and inflammatory activation. Activated microglia release proinflammatory cytokines (e.g., TNFα, IL1β, IL12, IFNγ), mitogenic factors (e.g., MCP-1, M-CSF), neurotrophic factors (e.g., IGF-1), and anti-inflammatory factors (e.g., TGF-β) [24], all of which may exert a neurotoxic effect on the MNs. Cytokines secreted by activated microglia, including IL1α, TNFα and C1q, have been shown to further induce the A1 subtype of reactive astrocytes (key in neuronal death in neurodegenerative diseases including ALS) [25]. Moreover, the levels of different cytokines, such as G-CSF, IL2, IL15, IL17, MCP-1, MIP1α, TNFα, and VEGF, have been identified in abnormally high levels in the cerebrospinal fluid (CSF) of patients with ALS [26]. Moreover, IL-6 levels in astrocyte-derived exosomes were also found increased in SALS patients, thus suggesting that CNS-derived exosomes could be useful to reveal neuroinflammation of the CNS in ALS patients and positively associated with the rate of disease progression [27]. All these phenomena further implicate neuroinflammatory processes in the pathogenesis of ALS. Importantly, recent data demonstrate that neuroinflammation is linked to the symptomatic phase of ALS/FTD (frontotemporal dementia) and shows a similar pattern in sporadic and genetic cases. ALS and FTD are characterized by a different neuroinflammatory profile, which might be one driver of the diverse presentations of the ALS/FTD syndrome [28].

In spite of the mounting evidence uniting neuroinflammatory processes and neuronal death in ALS, the immune system appears to be protective during the early stages of the disease. Glia and T cells, especially M2 macrophages/microglia, and T helper (Th) 2 cells and regulatory T (Treg) cells are key anti-inflammatory factors that sustain MN viability. However, as the disease progresses and MN injury accelerates, a second rapidly-progressing phase develops, that is characterized by increased levels of M1 macrophages/microglia, and proinflammatory Th1 and Th17 T cells [29]. It has been suggested that the neuroprotective M2/Treg/Th2-mediated pathways are downregulated whereas the cytotoxic M1/Th1/Th17 pathways are upregulated, resulting in a self-propagating proinflammatory acceleration of disease progression [30,31,32,33,34].

Neuroinflammation and oxidative stress (OS) are inextricably linked in the pathogenesis of neurodegenerative diseases. OS is the consequence of increased production of reactive oxygen species (ROS), which is frequently accompanied by a decrease in antioxidant defenses [35]. Glial and infiltrated immune cells are considered among the major producers of ROS and reactive nitrogen species (RNS) in pathological conditions of the CNS [36]. Although ROS are not believed to cause ALS, they are likely to exacerbate disease progression [17].

OS may also contribute to the degeneration of the neuromuscular junction in ALS. Increased sensitivity of the nerve terminal to ROS has been shown in ALS mouse models, which may facilitate presynaptic decline in neuromuscular junctions. Moreover, excitatory amino acid-mediated overstimulation of MNs results in abnormal secretion of acetylcholinesterase, which will decrease the acetylcholine present in the synaptic cleft (a mechanism that could be behind the loss of muscle strength observed in ALS patients). These early-stage dysfunctions are accompanied by inflammatory agents and loss of trophic support, which ultimately may lead to neurodegeneration [37].

There is also evidence that the response to OS is dampened in ALS. Levels of glutathione (GSH), a prevalent antioxidant in mammalian cells, are lower in the motor cortex of ALS patients as compared to healthy volunteers [38,39]. Moreover, expression of mutant TDP-43 in a MN-like cell line induces OS and mitochondrial damage eliciting nuclear accumulation of nuclear factor E2-related factor 2 (Nrf2), a master regulator of detoxification and also of antioxidant, anti-inflammatory and other cytoprotective mechanisms [40,41]. Dysregulated Nrf2-dependent antioxidant pathways have been implicated in ALS, and therapeutic strategies targeting the Nrf2 antioxidant response element are under investigation [42]. Nrf2 mRNA and protein levels are depleted in post-mortem tissues from ALS patients [42], and studies in ALS mouse models have shown a significant beneficial effect of elevated Nrf2 levels in astrocytes, the major GSH suppliers for neighboring neurons [43], Furthermore, Nrf2 signaling is critical for attenuating neuroinflammation in ALS through repression of the deleterious effects of activated microglia on neurons [44].

From all this evidence, we can conclude that neuroinflammation and OS are intertwined mechanisms involved in the physiopathology of ALS.

## 3. Mitochondria and the Mechanism of Motor Neuron Death

Mitochondrial dysfunction has also been implicated as a key neuropathological hallmark of ALS [12]. For instance, reduction of the activity of the electron transport chain complexes (complex I–IV) in post-mortem spinal cord specimens from SALS patients [45], reduced intracellular ATP levels in lymphocytes of ALS patients [46], or inhibition of the voltage-dependent anion channel (VDAC) by mutant SOD1 [47].

The class III deacetylases, or sirtuins (SIRT), are a family of conserved epigenetic mediators that regulate a myriad of cellular processes relating to health- and life-span. Three of the seven known SIRT (SIRT3, SIRT4 and SIRT5) are primarily localized to the mitochondria [48].

SIRT3 has been extensively studied for its role in energy homeostasis. In addition to deacetylating and regulating several mitochondrial enzymes (such as acetyl-CoA synthase 2 and glutamate dehydrogenase, SIRT3 has been shown to regulate the production of mitochondrial ROS [49].

SIRT1 is primarily nuclear and has been shown to regulate longevity by maintaining mitochondrial homeostasis and mitophagy. Interestingly, intraperitoneal injection (but not oral administration) of resveratrol, a potential SIRT1 activator, led to a significant improvement in both symptoms and survival in the SOD1^G93A^ mouse model of ALS [50]. This is consistent with recent findings where overexpression of SIRT1 in MN of SOD1^G93A^ mice delays the onset of the disease and increases survival [51]. Moreover, SIRT1 may have wide-reaching implications on mitochondrial biogenesis via the deacetylation of PGC-1α [52]. PGC-1α is downregulated in SALS patients [53]. A decrease in PGC-1α associates with lower expression of SIRT3, a regulator of mitochondrial energy metabolism and ROS that is regulated by PGC-1α [54]. Overexpression of SIRT3 in cultured cells increases respiration and PGC-1α expression, and decreases the production of ROS [54,55]. Furthermore, overexpression of SIRT3 and PGC-1α protects against mitochondrial fragmentation and death in SOD1^G93A^ MNs [54]. Moreover, SIRT3 deficiency results in hyperacetylation of SOD2 and cyclophilin D, two mitochondrial proteins involved in regulating adaptive responses of neurons to physiological challenges and resistance to degeneration. SIRT3 was also shown to regulate ketone body production by deacetylating mitochondrial 3-hydroxy-3-methylglutaryl CoA synthase 2. This evidence is consistent with the elevation of mitochondrial SIRT3 expression in medium chain triglyceride-treated primary MN cultures and in the spinal cord of SOD1^G93A^ mice following medium chain triglyceride treatment [56]. The authors hypothesize that medium chain triglycerides might regulate mitochondrial activity and cell survival through sirtuin-mediated responses.

Consumption of NAD^+^ without an adequate method of replenishment can result in decreased activity of SIRT-dependent processes, and a consequential deleterious effect on mitochondrial biogenesis, function and mitophagy [48]. Thus, SIRT deactivation may contribute to the mitochondrial dysfunction associated with ALS. Indeed, it has been shown that methods to enhance NAD^+^ salvage pathways were able to attenuate the neurotoxic phenotype of SOD1^G93A^ astrocytes; this observation was most likely due to increased SIRT1 and SIRT3 activities associated with greater NAD^+^ availability [57]. Thus, methods to increase SIRT activity, by restoring NAD^+^ levels or via direct activation using small molecules, are potential therapeutic approaches in the treatment of ALS.

A recent human pilot study [58], demonstrating that the experimental therapeutic EH301 may be able to slow the progressive decline in functionality, strength and lung function, may offer new insight into the pathophysiology underpinning ALS. EH301 is a combination of nicotinamide riboside (NR) and pterostilbene (PT); compounds that are predicted to increase availability of the coenzyme NAD^+^ and support the activity of the SIRT and their downstream targets.

NR, a pyridine-nucleoside form of vitamin B3, is a precursor to NAD^+^ with superior pharmacokinetic profile relative to other forms of B3 (nicotinic acid and nicotinamide) [59]. Since NAD^+^ depletion associates with neuronal death [60], it has been proposed that increasing NAD^+^ availability may be neuroprotective [61]. Gerdts et al. demonstrated, using a model of sterile alpha and TIR motif-constraining 1 (SARM1)-induced axonal death, that NAD^+^ levels are rapidly depleted following axonal injury [62]. Treatment with NR was shown to prevent axonal degeneration and cell death in this model.

Additionally, two modes of operation by isolated mitochondria result in significant O_2_^•−^ production, predominantly from complex I: (i) when the mitochondria are not making ATP and consequently have a high Δp (protonmotive force) and a reduced CoQ (coenzyme Q) pool; and (ii) when there is a high NADH/NAD^+^ ratio in the mitochondrial matrix. For mitochondria that are actively making ATP, and consequently have a lower Δp and NADH/NAD^+^ ratio, the extent of O_2_^•−^ production is far lower [63]. NAD^+^-derived nicotinamide suppresses O_2_^•−^ generation via reduction of electron transport and increases membrane potential (Δψm) via downregulation of mitochondrial permeability transition pore (mtPTP) formation [64].

PT (3,5-dimethoxy-4′-hydroxystilbene) is a naturally occurring stilbenoid, found primarily in blueberries and *Pterocarpus marsupium* heartwood. PT is a dimethylated analogue of resveratrol; a SIRT1-activating molecule that has been shown to increase lifespan in different model organisms [65]. Han et al. reported delayed disease onset and increased survival in resveratrol-treated SOD1^G93A^ mice [50]. The authors suggested that the therapeutic benefits of resveratrol may be due to increased SIRT1 activation, which was measured via deacetylation of p53 (a major SIRT1 substrate). Mancuso et al. and Song et al. also reported delayed disease onset, extended survival and improved spinal motor neuron function in SOD1^G93A^ mice, and both authors noted increased SIRT1 activation in the spinal cord [66,67]. However the low bioavailability of this polyphenol has limited its translation into the clinical setting [65]. PT substitutes two hydroxyl groups in resveratrol for methoxy groups, modifications that increase PT’s lipophicity and half-life. PT can cross the blood brain barrier and has been shown to increase nuclear Nrf2, thus potentially promoting the antioxidant defenses in the CNS [68]. Interestingly, PT has also been shown to attenuate early brain injury following subarachnoid hemorrhage via inhibition of the nucleotide-binding oligomerization domain-like receptor family pyrin domain-containing 3 (NLRP3) inflammasome and NOX2-related OS [69]. Which may further support the therapeutic role of PT in ALS, since the microglial NLRP3 inflammasome is activated by ALS proteins [70].

**Figure 1:**
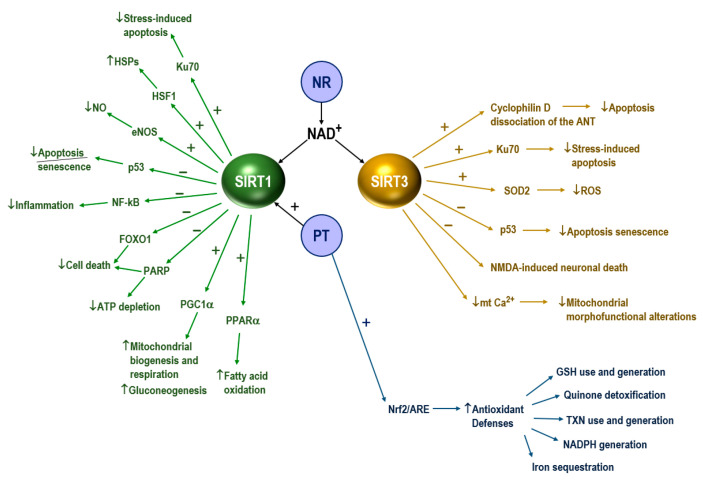
Molecular interactions underlying the effect of nicotinamide riboside (NR) and pterostilbene (PT) in amyotrophic lateral sclerosis (ALS). SIRT1, sirtuin 1; SIRT3, sirtuin 3; HSPs, heat-shock proteins; HSF1, heat-shock factor 1; NF-kB, nuclear factor kappa-light-chain-enhancer of activated B cells; FOXO1, forkhead box protein O1; PARP, poly (ADP-ribose) polymerase; PGC1α, peroxisome proliferator-activated receptor gamma coactivator 1-alpha; LXRs, liver X receptors; eNOS endothelial nitric oxide synthase; PPARα, peroxisome proliferator-activated receptors alpha; Nrf2/ARE, transcription factor Nrf2 (NF-E2-related factor 2)/antioxidant responsive element; ANT, adenine nucleotide translocator; SOD2, superoxide dismutase 2; NMDA, N-metil-D-aspartato; GSH, glutathione; TXN, thioredoxin. NR increases the availability of NAD^+^, which supports the activity of SIRT1 and SIRT3 and their downstream targets. PT activates SIRT1 and Nrf2. Activation of Nrf2 in MNs results in the induction of many cytoprotective proteins, i.e., (but are not limited to) the following: (1) GSH use and generation (γ-glutamyl-cysteine synthase, GSH reductase, xCT (a component of the cysteine/glutamate transporter), GSH peroxidase 2, different GSH transferase isoenzymes); (2) quinone detoxification (NAD(P)H dehydrogenase (quinone 1)); (3) thioredoxin use and generation (thioredoxin 1, peroxiredoxin 1, thioredoxin reductase 1); (4) iron sequestration (heme oxygenase 2); (5) NADPH generation (glucose-6-P dehydrogenase, phosphogluconate dehydrogenase, malic enzyme 1, isocitrate dehydrogenase 1) [42,65,73,74,75,76,77,78,79].

Figure 1 displays an integrated scheme of the molecular interactions underlying the potential benefits exerted by the combination of NR and PT. mtGSH depletion (either by oxidation or by inhibition of its transport) can promote the release of proapoptotic signals to the cytosol [71]. In this regard Mytilineou et al. provided evidence that neuronal GSH depletion in the presence of glial cells leads to neuronal degeneration [72].

**Figure 2:**
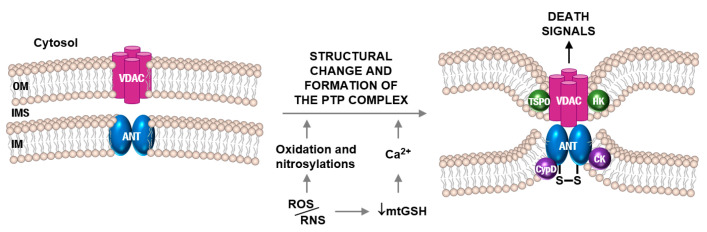
Opening of the mtPTP complex preceding apoptotic motor neuron death during ALS pathophysiology. The voltage-dependent anion channel (VDAC) and mitochondrial adenine nucleotide translocator (ANT) are both dimers located in the outer and inner membranes, respectively. High membrane potential in normally functioning mitochondria keeps the mitochondrial permeability transition pore (mtPTP) closed. Under pathophysiological conditions such as oxidative/nitrosative stress, the mtPTP opens, allowing the entry of H_2_O and solutes. Thus, causing mitochondrial swelling and release of apoptosis-initiating factor (AIF) and cytochrome c from the intermembrane space (IMS), which ultimately results in apoptosis. ROS/RNS cause mtGSH depletion and, consequently, higher levels of intramitochondrial free Ca^2+^, oxidation of critical redox-sensitive –SH groups of the ANT (e.g., Cys56; which, under physiological conditions, are in equilibrium with matrix GSH), and decreased mitochondrial membrane potential. All these factors can trigger structural changes leading to the formation of the mtPTP complex. Translocator protein (TSPO), hexokinase (HK), creatine kinase (CK), and cyclophilin D (CypD) are the other components of mtPTP. Outer membrane (OM), inner membrane (IM).

The role of the adenine nucleotide translocator (ANT) in apoptosis is well known [80] and, as part of the mechanisms linking apoptosis and ALS, the mtPTP has been implicated in the pathogenesis [81]. The ANT forms the inner membrane channel of the mtPTP complex [82]. In vitro cell experiments have shown that NO and ONOO^-^ can act directly on the ANT to induce mitochondrial permeabilization [83]. Interestingly the ANT and its regulator (cyclophiline D, CyPD) were found as targets of nitration in ALS mice [84]. SOD1^G93A^ mice without CyPD (genetic ablation of the *Ppif* gene) showed markedly delayed disease onset and lived significantly longer than mice with CyPD [84]. In ALS patients affected anterior horns of the spinal cord are depleted of large MN, whereas the remaining MN are partially atrophic [81]. Cell death assays identified subsets of MN in the process of DNA fragmentation as the nucleus condenses and the cell body shrinks [81]. Moreover, in ALS patients, p53 (which promotes apoptosis via the Apaf-1/caspase-9 pathway and involves mitochondrial cytochrome c release) [85], accumulates in the nucleus of MN. Based on these observations and related background a plausible model of the mechanism leading to apoptotic cell death in MN is displayed in Figure 2.

Furthermore, it has been shown that nuclear protein TDP-43 (hyper-phosphorylated and ubiquitinated TDP-43 deposits act as inclusion bodies in the brain and spinal cord of SALS patients) also interacts with mitochondrial proteins critical for mitochondrial dynamics and mitophagy, including voltage-gated anion channel 1 (VDAC1) and prohibitin 2 (PHB2), a crucial mitophagy receptor [86]. Moreover, genetic studies (i.e., gene mutations affecting DCTN1, OPTN, TBK1, VCP, and C9ORF72) have implicated deficits in autophagy and/or mitophagy in the onset of the disease [87]. Thus, it is plausible that altered selective sequestration and subsequent degradation of the dysfunctional mitochondrion, before it causes activation of cell death, may contribute to the irreversible death of MNs. In this regard, it has been reported that ALS-FTD-linked mutations of SQSTM1/p62 disrupt selective autophagy and NRF2-dependent anti-OS mechanisms [88]. Which further suggests links between autophagy/mitophagy and OS in the pathophysiology of ALS.

**Figure 3:**
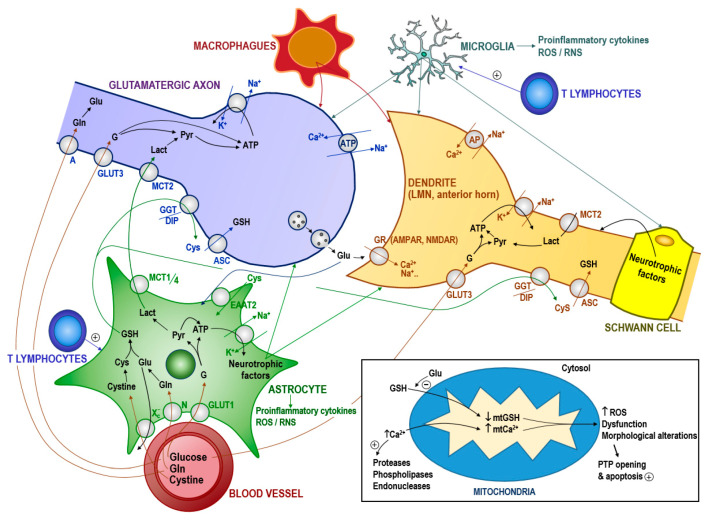
Fluxes of metabolites in the microenvironment of the motor neuron and the activation of mitochondria-dependent apoptosis. We can hypothesize that RNA-protein associations could theoretically be the starting point of protein misfolding leading to insoluble structures (e.g., of TDP-43) which could eventually associate to (and recruit) other native proteins. This progressive mechanism, which resembles the typical prion replication, may lead to the formation of abnormal protein complexes spreading (in the case of ALS) throughout the MN network. Interestingly some recent findings suggest that extracellular vesicles may facilitate the spread of toxic proteins and might play a role in the prion-like propagation of ALS disease [92]. Nevertheless, a mechanism mediated by a (food related?) neurotoxin cannot be ruled out. Abnormal protein- or neurotoxin-induced alteration of the interaction between glutamate and its receptor could cause glutamate accumulation in the synaptic cleft (and also within the presynaptic glutamatergic neuron?). This accumulation could be exacerbated by defects in astrocyte glutamate uptake due to loss of the EAAT2 transporter occurring in both SALS and FALS cases [93,94]. In fact, glutamate induces far greater ROS generation in cultured MNs than in other spinal neurons. Hypothetically, in the MNs a glutamate-induced inhibition of GSH uptake by mitochondria coupled with proinflammatory cytokines, such as TNFα, could promote an increase in ROS, thereby leading to mitochondrial dysfunction and activation of cell death in a mechanism similar to that described in tumor cells [90]. Activated microglia and astrocytes express and release high levels of TNFα [95]; these events would then activate a (more intense) local inflammatory response involving T lymphocytes, activated microglia and macrophages. Inflammation-associated alteration of the ionic equilibrium would favor Ca^2+^ influx, which would further promote mitochondrial dysfunction. Glutamate accumulation in the extracellular space would also affect cysteine uptake and, thereby, GSH synthesis in astrocytes and its export to neurons [96]. Intracellular GSH depletion facilitates the release of Ca^2+^ from intracellular deposits [97]. ROS (and associated RNS) could also damage different transport mechanisms such as glucose transporters. ROS/RNS-induced protein damage would also favor the formation of protein aggregates and cause impaired axonal transport. Any exogenous pro-oxidative stress factor (e.g., radiations, metal toxicity, intense physical activity, or low intake of dietary antioxidants) could aggravate the progression of the disease. Moreover, as the disease progresses, nutritional deficiency, cachexia, psychological stress, and impending respiratory failure may further increase oxidative stress leading inexorably to MN death. GR, glutamate receptors; AMPAR, α-amino-3-hydroxy-5-methyl-4-isoxazolepropionic acid receptor; NMDAR, N-methyl-D-aspartate receptor; ROS/RNS, reactive oxygen and nitrogen species; GLUT1, glucose transporter 1; GLUT3, glucose transporter 3; MCT, lactate transporter; EATT, excitatory amino acid transporter; N and A, Gln transporters; Xc^-^, cysteine/glutamate antiporter; ASC, transport system for neutral amino acids; AP, Ca_2_^+^/Na^+^ antiporter; GGT/DIP, γ-glutamyl transpeptidase and dipeptidases; mt, mitochondrial. PTP, permeability transition pore complex; G, glucose; Pyr, pyruvate; Lact, lactate; GSH, glutathione; Cys, cysteine; Gln, glutamine; Glu, glutamate.

The ‘glutamate hypothesis’, that implicates glutamate-induced cytotoxicity, remains a primary theory of the mechanism leading to MN death. Glutamate is synthesized from glutamine by the enzyme glutaminase. This can occur in the presynaptic neuron or in neighboring glial cells. The cytosolic concentration of glutamate in glutamatergic neurons is in the mM range [89]. However, the intracellular glutamate concentration in the MNs of patients or mouse models of ALS under in vivo conditions is unknown; and it is also unknown if it is of any relevance. Glutamate is a competitive inhibitor of GSH transport into mitochondria [90], and mtGSH depletion and Ca^2+^ load could initiate the cascade of events causing MN death, including mitochondrial dysfunction, oxidative/nitrosative stress-associated damage, formation of Ca^2+^-rich precipitates, and the release of proapoptotic signaling molecules to the cytosol (Figure 3). Preliminary experiments performed in our lab show that MNs isolated from FUS R521C transgenic mice (which develop robust neuronal loss in the spinal cord, denervation of neuromuscular junctions, and muscle atrophy) [91] show mtGSH depletion and higher cytosolic glutamate levels as compared to normal wild-type C57BL/6 mice (Table 1) (Estrela et al., unpublished results). This potential mechanism and the underlying metabolic adaptations, and whether apoptosis is the preferent type of MN death in ALS, are under investigation in our laboratories. Figure 3 schematically outlines the main metabolite flows in the MN microenvironment.

## 4. Implications in the Therapy of ALS

Riluzole (shown to compensate for harmful extracellular glutamate levels), currently prescribed to most ALS patients, has been found to improve survival by 2–3 months [103]. In addition to its role in accelerating glutamate clearance from the synapse, riluzole may also prevent glutamate release from presynaptic terminals [104] and therefore could favor pathophysiology (Figure 3). Edaravone (an antioxidant) was approved for the treatment of ALS in the US in 2017 based on the results from a randomized placebo-controlled clinical trial in subjects with early-stage ALS in Japan (NCT01492686, www.clinicaltrials.gov). In this trial edaravone significantly slowed the 24-week decline in functionality, measured via the revised ALS functional rating scale (ALSFRS-R), in the edaravone-treated group relative to placebo (−5.01 compared to −7.50, respectively). However, edaravone has failed to demonstrate efficacy in two trials in patients with all stages of ALS, and may not be a viable treatment option for the wider patient population [105,106].

Other therapeutic approaches under consideration have been recently reviewed [107] and include: (a) masitinib [108] which demonstrated a 27% reduction in functional decline when administered along with riluzole in a 48-week placebo-controlled phase 3 trial. Masitinib, a tyrosine kinase inhibitor, is predicted to work by reducing microglia-based inflammation of MNs in the brain and spinal cord (NCT02588677, www.clinicaltrials.gov); (b) AMX0035, a combination of sodium phenylbutyrate and tauroursodeoxycholic acid, aimed to reduce nerve cell death by limiting cell death and neuroinflammation; (c) antisense therapy; (d) gene therapy; or (e) stem-cell therapy using mesenchymal stromal cells isolated from e.g., adipose tissue. Mesenchymal stromal cells secrete small proteins (e.g., neurotrophic factors) that support the survival of motor neurons and also support the immune system, which may be relevant given a role for the immune system and its associated inflammation in ALS. At present all other clinical procedures used in ALS patients are essentially palliative: breathing support, physical therapy, and end of life care.

Based on available evidences and the pathophysiology mechanisms described above, we might suggest other options to be also explored, i.e., therapeutic agents to control the intracellular glutamate cytotoxicity (e.g., necrostatin-1) [109,110]; anti-inflammatory drugs [111,112]; specific cannabinoid receptors such as CB2 (to improve neuroprotection) [113]; metal chelators, e.g., the iron chelator deferiprone (to limit metal-induced toxicity leading to OS) [114]; neurotrophins (to promote neuroplasticity) [115]; cyclic nucleotide phosphodiesterase (PDE) inhibitors to prevent glial cell activation (e.g., ibudilast) [116]; antiretrovirals (e.g., TRIUMEQ^®^, a combination of abacavir sulfate, dolutegravir sodium, and lamivudine) used as an anti-HIV therapy, and based on the fact that ALS patients present serum concentrations of reverse transcriptase comparable to HIV-infected patients [117]; antiepileptic drugs (e.g., retigabine, which acts by binding to the voltage-gated K^+^ channels and increasing the M-current, thus leading to membrane hyperpolarization and decreasing excitability) [118]; or the antiestrogen tamoxifen (some neurological improvements have been observed in ALS patients with breast cancer and treated with tamoxifen—an effect possibly related to the inhibition of protein kinase C, which is overexpressed in the spinal cord of ALS patients) (see e.g., NCT00214110 under www.clinicaltrials.gov).

## 5. Concluding Remarks

In light of recent evidences highlighting the therapeutic potential of sirtuin activation and NAD^+^ repletion, a sequential cascade of events in the pathophysiology of ALS begins to be visualized.

Neuroinflammation, involving reactive astrocytes and microglia, and peripheral immune cells, promotes OS in the MN microenvironment. In this scenario many of the suggested causes/factors of SALS could be secondary, with the plausible exception of an infectious agent and/or an autoimmune mechanism. Neurodegeneration and the death of the MN would be the consequence of these initial phenomena. Experimental evidences suggest that damage to mitochondria could be a key mechanism involved in the final activation of neuronal death.

New therapeutic combinations could hopefully offer options to control the disease and extend the life expectancy of patients. In order to fulfil this aim, targeting patients early in their disease course may be critical. Finally, given the heterogeneity of disease progression, as well as key pathogenic differences according to disease stage, precision approaches combining genomics, biomarkers, imaging markers, and targeted/combined therapeutics may help to improve the clinical outcome.

## Figures and Tables

**Table 1:** ROS generation and levels of glutamate, GSH, GSSG and Ca^2+^ in motor neurons isolated from wild-type (WT) and FUS R521C mice.

	Motor Neurons					
	WT			FUS R521C		
	cyt	mt	Total	cyt	mt	Total
**H_2_O_2_** (nmol/10^6^ cells·min)			0.42 ± 0.17			1.06 ± 0.33 **
**O_2_**^**•−**^ (ΔFL1, a.u.)			2.11 ± 0.66			4.33 ± 1.20 *
**Glutamate** (nmol/10^6^ cells)	28.5 ± 4.3	5.6 ± 1.4		41.7 ± 5.9 **	7.2 ± 1.8	
**GSH** (nmol/10^6^ cells)	21.2 ± 2.9	4.3 ± 1.2		15.0 ± 2.4 **	2.1 ± 0.5 **	
**GSSG** (nmol/10^6^ cells)	0.5 ± 0.2	0.2 ± 0.05		1.0 ± 0.3 *	0.4 ± 0.1 **	
**Ca**^2+^ (nmol/mg protein)	1087 ± 184	512 ± 96		1740 ± 260 **	826 ± 160 *	

High yield extraction and culture of pure spinal motor neurons (MNs) were performed as previously described [98]. Wild-type C57BL/6 and transgenic FUS R521C (expressing mutant FUS protein) mice were obtained from Jackson Labs. FUS R521C mice show, at postnatal day 60, about 50% loss of anterior horn neurons (remaining motor neurons show reduced dendritic complexity and synaptic density). MN isolation and measurements were performed at postnatal day 100. Cytosolic (cyt) and mitochondrial (mt) compartments were rapidly separated using digitonin and centrifugation through a layer of silicon oil, as previously described for other cell types [99]. Measurement of H_2_O_2_ based on the H_2_O_2_/horseradish peroxidase-dependent oxidation of homovanillic acid (3-methoxy-4-hydroxyphenylacetic acid) to a highly fluorescent dimer (2,2-dihydroxydiphenyl-5,5-diacetic acid) and flow cytometric determination of O_2_^−^ generation were performed as previously described (a.u., arbitrary units) [100]. Glutamate was measured fluorometrically by a standard enzymic method [101]. Glutathione (GSH) and glutathione disulfide (GSSG) levels were determined by liquid chromatography-mass spectrometry as previously described [102]. Free Ca^2+^ levels were measured using the Ca^2+^ indicator Fura-2 AM and following the methodology described by Abcam (Cambridge, UK). Results are expressed as means ± SD for 6–7 different experiments. * *p* < 0.05, ** *p* < 0.01 (Student’s *t*-test) comparing MNs from FUS R521C versus their equivalent wild-type.
